# Comparative Evaluation of the Effect of Two Platelet Concentrates (a-PRF and L-PRF) on the Cellular Activity of Pre-osteoblastic MG-63 Cell Line: An in vitro Study

**DOI:** 10.30476/dentjods.2022.93305.1709

**Published:** 2023-06-01

**Authors:** Azadeh Esmaeilnejad, Mohammadreza Talebi Ardakani, Mahdi Shokri, PhD; Nima Hosseini Khou, Mobina Kamani

**Affiliations:** 1 Dept. of Periodontics, School of Dentistry, Shahid Beheshti University of Medical Sciences, Tehran, Iran; 2 Dept. of Dental Biomaterials, School of Dentistry, Shahid Beheshti University of Medical Sciences, Tehran, Iran; 3 Private Practice, Tehran, Iran; 4 Postgraduate Student, Dept. of Periodontics, School of Dentistry, Guilan University of Medical Sciences, Rasht, Iran

**Keywords:** Platelet-Rich Fibrin, Bone Regeneration, Cell Proliferation, Cell Differentiation

## Abstract

**Statement of the Problem:**

Currently, the reconstruction of bone defects with new platelet concentrates is considered a significant challenge in periodontics.

**Purpose:**

This study aimed to evaluate advanced- platelet rich fibrin (A-PRF) and leukocyte- and platelet rich fibrin’s (L-PRF) effects on the proliferation and differentiation of MG-63 cells.

**Materials and Method:**

In this *in vitro* study, blood samples of five healthy non-smoking volunteers were collected and immediately centrifuged according to the two protocols of Choukroun and Ghanaati, without adding any anticoagulants, to prepare L-PRF and A-PRF. After freezing the clots for one hour, they were crushed and centrifuged once more. After culturing MG-63 cells, the effects of 20%, 10%, 1%, and 0.5% concentrations of A-PRF and L-PRF extracts on cell proliferation and mineralization were evaluated by methyl thiazolyl tetrazolium (MTT) assay and Alizarin Red staining, respectively.

**Results:**

Generally, survival and proliferation in the L-PRF group at both time intervals were higher than the A-PRF group and increased with increasing the extract concentration. However, in the A-PRF group, there were no significant differences between the different concentrations, and only the number of cells increased over time. After three days, in the study on mineralization, nodule formation was observed only in the positive control group (osteogenic). In seven days, mineralized nodules were formed in all groups with different concentrations of A-PRF, but not in any of the L-PRF groups.

**Conclusion:**

According to the results, L-PRF increased proliferation, and A-PRF exerted a positive effect on the differentiation of MG-63 cells.

## Introduction

Reconstruction of bone defects due to trauma, infection, tumor, and congenital lesions is a critical clinical problem. Despite the availability of allografts and xenografts, the use of these bone substitutes is limited due to the risk of host immune response and the possibility of pathogen transmissions. Fortunately, new advances in tissue engineering techniques have provided promising alternatives for bone defect reconstruction [ 1 ]. Tissue engineering requires a cellular scaffold that provides the physiological state where bone cells can migrate, proliferate, and differentiate to form new bone [ 2 ]. Accordingly, the role of growth factors in wound healing and periodontal tissue regeneration is critical [ 3 ].

The second generation of platelet concentrates, called Choukron’s platelet rich fibrin (PRF) or leukocyte- and platelet rich fibrin (L-PRF), was introduced in 2006 [ 4 ]. Due to the lack of any anticoagulants and biochemicals in PRF preparation, physiological polymerization of fibrin provides a network for cellular migration and proliferation, and thus for wound healing.

Activation and degranulation of platelets and leukocytes during centrifugation results in release of cytokines and growth factors from L-PRF significantly during fibrin matrix remodeling. In addition to inflammation regulation [ 5 - 6 ], L-PRF serves as a reservoir of the most significant growth factors, such as platelet-derived growth factor (PDGF), transforming growth factor beta (TGFβ), and insulin-like growth factor (IGF) [ 3 , 5 - 7 ]. These factors accelerate the cellular cycle, activate angiogenesis, speed up scar tissue formation and wound closure, and cause healing without infection [ 8 ]. Furthermore, using L-PRF does not require exogenous scaffolds as a carrier for cells [ 1 ]. The PRF provides an appropriate scaffold that maintains cytokines and facilitates osteogenic differentiation [ 9 - 10 ]. L-PRF exudates also enhance the proliferation and osteogenic differentiation of periodontal ligament cells *in vitro* [ 11 ]. Wang *et al*. [ 12 ] showed that the application of L-PRF with mesenchymal stem cell sheets resulted in the healing of rabbit’s calvarial bone lesions in critical size.

In 2014, Ghanaati *et al*. [ 13 ] introduced a new product called advanced- platelet rich fibrin (A-PRF). They found that a change in centrifugation force (reducing revolutions per minute and increasing time) in the preparation of L-PRF resulted in a better distribution pattern of monocytes and B and T cells in the whole membrane. The A-PRF releases a large amount of TGFβ, IGF, PDGF, and epidermal growth factor in 10 days, and also exhibits high levels of and collagen type I mRNA [ 14 ]. A-PRF has a higher content of PDGF, TGFβ and vascular endothelial growth factor (VEGF) than other platelet concentrates like platelet-rich plasma, concentrated growth factor, and plasma rich in growth factor and significantly induces in vitro proliferation in human periosteal cells [ 15 ]. It is also indicated that the A-PRF membrane has the highest ability to release TGFβ, PDGF, VEGF, and epidermal growth factor for 10 days compared to the L-PRF [ 16 ].

Considering the uniform, continuous, and long-term release pattern of growth factors [ 17 ], the A-PRF can be used as an appropriate membrane to improve bone marrow proliferation and differentiation. Moreover, A-PRF alone or in combination with freeze-dried bone allograft compared to freeze-dried bone allograft alone was a more suitable biomaterial for ridge preservation; the amount of live bone volume and mineral density in samples of patients treated by A-PRF was significantly higher, and ridge height reduction was low [ 18 ]. However, contradictory results have been reported in another study [ 19 ].

Increasing the clinical use of platelet membranes (like L-PRF and A-PRF) for improving bone remodeling around teeth and implants as well as their easy application would justify the need for further studies on these platelet products. Due to the lack of laboratory information about their effects on osteoblasts, this *in vitro* study aimed to evaluate the influence of different concentrations of A-PRF and L-PRF extracts on the cellular activity of pre-osteoblastic MG-63 cells for the first time. 

## Materials and Method

This randomized controlled single-blind *in vitro* trial was conducted to evaluate the effect of A-PRF and L-PRF extract on proliferation and mineralization of MG-63 cells. The study population was pre-osteoblastic MG-63 cells that were cultured in cell culture plates with an initial density of 50,000 cells.

### Preparation of cells

The MG-63 cells (osteosarcoma cell line, human osteoblast-like cells) were purchased from the Pasteur Institute Cell Bank (Code C555). The cells were cultured in 75-mL flasks in Dulbecco’s Modified Eagle Medium (Gibco, USA) containing 10% fetal bovine serum (FBS), penicillin 10 IE /L, and streptomycin 100µg /L at 37°C in an incubator (C150, Binder, Germany) with 95% air, 5% CO_2_ and 90% humidity. 

The cells were passaged every three days, and when cell confluency reached approximately 80%, they were detached from the flasks by trypsin-EDTA and counted under an optical microscope using trypan-blue dye and a Neubauer plate. Then the cells were divided into two groups. A group with a density of 3000/well was transferred to two 96-well plates (each containing 200 µL) to assay cell proliferation at 24 and 72 hours. The other group was transferred to two 24-well plates with a density of 30,000/well for three days and 10,000/well for seven days for histological evaluation of calcified nodule formation using Alizarin red staining. The cells were incubated for 24 hours to achieve monolayer cell growth and then washed with phosphate-buffered saline solution (Gibco, USA). The previous culture medium was replaced with a 1% FBS medium for 24 hours (starving stage) [ 20 ]. All the steps were performed under sterile conditions and a biological hood (Laminar Flow, Class II, Arster, Iran). 

Finally, the experimental groups were divided in terms of the culture medium. Control groups included negative control group of methyl thiazolyl tetrazolium (MTT) test (cell culture medium containing 10% FBS), positive control group of MTT test (distilled water), negative control group of viability and mineralization tests (cell culture medium containing 1% FBS), and positive control group of mineralization (osteogenic environment). The experimental groups consisted of 8 groups with concentrations of 20, 10, 1 and 0.5% of L-PRF or A-PRF extract in cell culture medium containing 1% FBS.

### Preparation of L-PRF and A-PRF extracts

Five nonsmoker volunteers (three females and two males) aged 20–40 years with systemic health, especially in terms of coagulopathy, diabetes, leukemia, and other blood conditions, and normal platelet and leukocyte count and normal coagulation time (including bleeding time, prothrombin time, and partial thromboplastin time), by simple non-random sampling method, were included in the study. The subjects had no history of taking drugs with effects on platelets or other blood cells, such as aspirin, in the last two weeks.

After explaining the study procedure and obtaining informed consent forms, 50 samples of 9 mL blood from the antecubital vein of these individuals (under sterile condition) were placed in 10-mL dry glass-coated plastic tubes (Blood collecting tube, Intra-spin, Intra-lock, USA), which are specialized for PRF preparation. The tubes were randomly (coin toss) divided into L-PRF and A-PRF after collection. The tubes were centrifuged according to the Choukroun [ 4 ] (2700 revolutions per minute, 12 minutes) and Ghanaati [ 13 ] protocols (1500 revolutions per minute, 14 minutes) for L-PRF and A-PRF preparation, respectively, without adding any anticoagulant. Then they were transferred under a biosafety cabinet. The fibrin clots of L-PRF and A-PRF were held with a forceps, cut off by scissors from above the red corpuscles layer, and transferred to the PRF box. It was noted that the buffy coat part did not separate from the fibrin clot.

The clots were converted to membranes in the PRF box after the serum disappeared. Then, the L-PRF and A-PRF membranes were separately frozen within the nitrogen tank for one hour at -80°C. After one hour, the frozen membranes were cut to small pieces by surgical blades and sterile scissors, and were centrifuged again (400g, 10min). The upper fluid of tubes (PRF extract) was collected, and 20%, 10%, 1%, and 0.5% concentrations of A-PRF and L-PRF extracts were prepared in Dulbecco’s modified eagle medium containing 1% FBS [ 15 ].

### Viability and cell proliferation

MTT assay was used to evaluate the effect of different concentrations of L-PRF, A-PRF extracts (20%, 10%, 1%, and 0.5%) on cell viability, and proliferation compared to describe positive and negative control groups [ 20 ]. After the cells starved in 1% FBS for 24 h, the triplicated test was performed by one individual at specified times. The cells were exposed to different concentrations of L-PRF and A-PRF for 24 and 72 hours. The-n, 96-well plates were retrieved from the incubator at each time, and after observation of the cells under an inverted microscope (Nikon, TE300, Japan), the medium was removed and replaced with a yellow solution of MTT (0.5mg MTT per 1mL of Dulbecco’s modified eagle medium) after washing the cells with phosphate-buffered saline solution. Finally, the plates were returned to the incubator and incubated for three hours at 37°C.

After confirming the formation of formazan crystals under the inverted microscope, the wells were evacuated and replaced with 100 microliter of dimethyl sulfoxide solvent until the crystals were dissolved and the purple color appeared. Next, the 96-well plate was mounted on an ELISA reader (Anthos, 2020, Austria), and spectrophotometric intensity absorption of resuscitated MTT was read at 570 and 620 nm. Finally, cell viability evaluation was performed by SPSS software (version 25.0, IBM, USA) and two-way analysis of variance and the statistical significance level was considered *p*< 0.05.

### Examination of mineralization (Alizarin Red staining)

Differentiation evaluation was performed by observing the formation of mineralized nodules with Alizarin Red staining. The cells were placed in two 24-well plates and exposed to 20%, 10%, 1%, and 0.5% PRF extracts and negative and positive control groups (culture medium containing 1% FBS and osteogenic medium, respectively) for three and seven days. The test was performed in duplicate. After evacuating the medium, the cells were washed with phosphate-buffered saline solution and fixed with cold ethanol for an hour, and then washed twice with water for five minutes each time. After washing, 2% Alizarin Red solution was placed on the cells for 30 minutes at room temperature. The colored solution was then removed, and the cells were rinsed with water four times for five minutes each time. The plates were investigated carefully under an inverted optical microscope at 20× and 40× magnifications to evaluate the cells’ morphology and detect mineralized nodules colored red to orange. The results of this staining examination were qualitatively reviewed and reported.

## Results

### Viability and cell proliferation evaluation by MTT assay

MTT assay was used to evaluate the effect of different concentrations (20%, 10%, 1%, and 0.5) of L-PRF and A-PRF extract on cell viability and proliferation. In this test, the cell viability in the negative control group (containing 1% FBS medium) was considered 100% and the average light absorption of each experimental group was expressed as a percentage compared to the control group (Table 1).

**Table 1:** Mean viability percentage of MG-63 cells in different experimental groups compared to the negative control group at 24 and 72 hours (A-PRF: Advanced- platelet rich fibrin, L-PRF: Leukocyte- and Platelet rich fibrin, FBS: Fetal bovine serum)

Time	Groups	%	Mean	Standard deviation
24 hours	A-PRF	20	103.00	21.00
		10	134.00	13.45
		1	131.33	20.03
		0.5	106.33	29.56
	L-PRF	20	128.33	30.98
		10	180.00	27.51
		1	142.00	0.00
		0.5	125.00	9.64
	FBS	10	104.67	26.35
		1	99.33	12.85
	Water	-	3.00	1.00
72 hours	A-PRF	20	330.33	24.70
		10	323.33	18.47
		1	365.33	100.10
		0.5	350.67	61.53
	L-PRF	20	826.33	21.22
		10	711.00	128.71
		1	478.33	49.13
		0.5	433.67	92.04
	FBS	10	372.33	23.58
		1	101.33	11.01
	Water	-	15.67	6.11

In both groups, the cell proliferation at 72 hours was greater than 24 hours. Compared to the control group, the average percentage of cells after 72 hours (392±8) was more than 24 hours (114±8). It means that the influence of time on the amount of proliferation in 72 hours was significantly higher than 24 hours (*p*< 0.001). 

In each experimental group, except for the control groups containing water and 1% FBS medium, the increase in the number of cells in 72 hours was significantly higher than 24 hours (*p*< 0.001). However, the effect of A-PRF and L-PRF on MG-63 cells’ proliferation in 24 hours was not significant (*p*> 0.05). Two-by-two comparison of different concentrations of the A-PRF and L-PRF extracts, showed no significant differences between the two extracts in the stimulation of cell proliferation during 24 hours between any of the concentrations (*p*> 0.05). However, at 72 hours, at 10% and 20% concentrations, the proliferation of the cells in the L-PRF group was significantly higher than the A-PRF group (*p*< 0.001), but it was not significant (*p*> 0.05) at other concentrations (1%, and 0.5%). 

At 72 hours, the highest rate of cell proliferation was observed in the 20% (826.33±21.22) and 10% (711.00± 128.71) L-PRF groups, with no significant difference between them (*p*> 0.05). 

The lowest growth of MG-63 cells was reported in the 10% A-PRF group (32.4± 8.47), which was significantly lower than the 1% (*p*< 0.05), 10% (*p*< 0.001), and 20% (*p*< 0.001) L-PRF groups, but there were no significant differences between any of the A-PRF concentrations (*p*> 0.05). 

**Figure 1:**
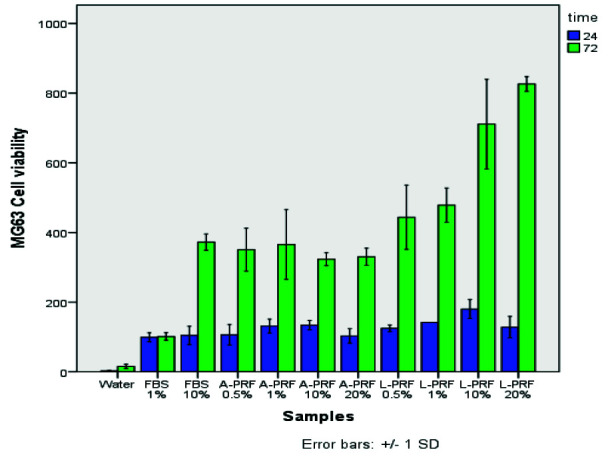
Proliferation assessment of MG-63 cells in different experimental conditions. Data are the mean±SD (A-PRF: Advanced- platelet rich fibrin, L-PRF: Leukocyte- and Platelet rich fibrin, FBS: Fetal bovine serum)

Generally, viability and proliferation in the L-PRF group at both time intervals were higher than the A-PRF group and increased with an increase in the concentration of extracts in the L-PRF group at 72 hours, with no change in the A-PRF group (Figure 1 and Table 2).

**Table 2:** The statistical significance, in comparing of the mean viability of MG-63 cells in the experimental and control groups at 24 and 72 hours based on MTT test, expressed in *p* Values (A-PRF: Advanced- platelet rich fibrin, L-PRF: Leukocyte- and Platelet rich fibrin, FBS: Fetal bovine serum)

		24 hours									
72 hours	A-PRF 20	1.000	1.000	1.000	1.000	1.000	1.000	1.000	1.000	1.000	0.655
	1.000	A-PRF 10	1.000	1.000	1.000	1.000	1.000	1.000	1.000	1.000	0.071
	1.000	1.000	A-PRF 1	1.000	1.000	1.000	1.000	1.000	1.000	1.000	0.087
	1.000	1.000	1.000	A-PRF 0.5	1.000	1.000	1.000	1.000	1.000	1.000	0.524
	0.000	0.000	0.000	0.000	L-PRF 20	1.000	1.000	1.000	1.000	1.000	0.109
	0.000	0.000	0.000	0.000	0.227	L-PRF 10	1.000	1.000	1.000	1.000	0.002
	0.019	0.011	0.268	0.092	0.000	0.000	L-PRF 1	1.000	1.000	1.000	0.038
	0.262	0.158	1.000	1.000	0.000	0.000	1.000	L-PRF 0.5	1.000	1.000	0.140
	1.000	1.000	1.000	1.000	0.000	0.000	0.437	1.000	FBS 10	1.000	0.586
	0.000	0.000	0.000	0.000	0.000	0.000	0.000	0.000	0.000	FBS 1	0.834
	0.000	0.000	0.000	0.000	0.000	0.000	0.000	0.000	0.000	1.000	Water

### Examination of mineralized nodules formation (Alizarin Red staining)

A qualitative study of mineralized nodule formation in MG-63 cells was performed by using Alizarin Red staining on days three and seven after exposure to different concentrations of A-PRF and L-PRF extracts. The plates were observed under an inverted optical microscope after preparation. After three days of exposure, no distinguished nodules were detected in any of the treated groups, whereas the ossified nodules were observed on day seven in all the A-PRF groups but not in any of the L-PRF groups.

## Discussion

Our results showed that only higher concentrations of L-PRF (10% and 20%) significantly increased the proliferation of MG-63 cells compared to other groups. In addition, seven days after exposure, only A-PRF (even at low concentrations) stimulated the production of calcium nodules. According to the results, these products might be used to repair bone defects.

The size of PRF clots created in this study varied in different individuals and even in several samples of one. Therefore, for standard comparison, it seems that using equal numbers of tubes from A-PRF and L-PRF and obtaining similar concentrations of extracts provide more similar conditions than using full-size clots with different sizes. In the present study, extracts were used instead of PRF membranes (by centrifuging for 10 minutes at 400 g force after shredding the membranes), which minimized the interfering factors useful in forming the membranes. 

In the present study, the MTT test showed that the rate of cell proliferation in the A-PRF group, despite the similar concentration, was less than half of that in the L-PRF group 72 h after exposure to extracts. This would possibly reflect the different characteristics of these two types of platelet products in growth factor levels, which

is influenced by the speed and time of centrifugation process. The reduction in relative centrifugal force, based on Choukroun *et al*. [ 21 ] research, generally resulted in a marked increase in leukocyte and platelet counts in the fibrin matrix and in Kobayashi *et al*. report [ 16 ] caused an increase in the secretion of growth factors, like PDGF, TGFβ, IGF, and VEGF, from A-PRF over L-PRF. 

In addition, we confirmed that L-PRF’s effect on the proliferation of MG-63 cells is dose- and time-dependent, consistent with other studies. As shown by Li *et al*. [ 22 ], the higher concentrations of L-PRF exudates at longer times lead to a more significant increase in the proliferation of PDL cells. In addition, in a similar study by Dohan *et al*. [ 23 ], the addition of two L-PRF membranes, compared with one, increased the proliferation rate of human mesenchymal stem cells at 14 days. In another study, Dohan *et al*. [ 24 ] showed that the effect of L-PRF on osteoblasts was always dose-dependent compared to other cells and consistently stimulated cell proliferation up to 28 days. This enhancing effect of L-PRF on proliferation can be justified by releasing PDGF and TGFβ, which have been shown to have mitogenic effects on cells [ 25 ].

In the present study, cell proliferation in the 20% L-PRF extract group was more than that in the 10% L-PRF group in 72 hours, but the differences were not statistically significant, which might be due to its dose-dependent effect on cell proliferation up to a certain dose of extracts. This has also been observed previously with other forms of platelet extracts. Graziani *et al*. [ 26 ] showed that optimum platelet-rich plasma’s platelet extract concentration affecting osteoblasts proliferation was 2.5 times over its blood concentration. Further studies should be carried out using higher L-PRF concentrations to obtain the maximum concentration of MG-63 proliferative platelet extract.

In the present study, the proliferation rate of MG-63 cells in the L-PRF group was significantly higher than that in the negative control group (10% FBS). Hence, it might be prudent to use lower concentrations of L-PRF extract instead of FBS in human cell proliferation studies, which results in eliminating the risk of infection and immune responses [ 27 ]. However, the possibility of its undesirable effects on the growth of cells should be considered.

In addition to evaluating cell proliferation, we studied the qualitative formation or non-formation of mineralized nodules in MG-63 progenitor cells, using Alizarin Red staining. Due to the absence of minerals in platelet extracts and cell culture medium used for dilution of extracts (Dulbecco’s modified eagle medium+1% FBS) and control groups, the observation of mineralized nodules in the medium can prove cellular secretion and might be a reason for the early differentiation of MG-63 cells. However, definitive evidence of bone differentiation must be established by examining the expression of early markers, such as alkaline phosphatase and collagen type I, to confirm the onset of osteoblast differentiation and quantify them over time to demonstrate the progression of osteoblast differentiation. The expression of secondary and late osteogenic markers such as osteopontin, osteonectin, and bone sialoprotein genes might also help establish active and mature osteoblasts from MG-63 preosteoblasts.

In the present study, the formation of mineralized nodules in the cells of all A-PRF groups after seven days showed that A-PRF generally stimulates the formation of mineralized nodules in MG-63 cells; however, at the same time, mineralized nodules were not formed in any of the L-PRF groups. This corresponds indirectly with the pattern of cell proliferation in the A-PRF and L-PRF groups since they coincide with the process of cellular development at times of proliferation and differentiation, and in reverse equilibrium [ 28 ]. Considering the results of the mineralization assay, it can be concluded that a part of the lower cell proliferation rate in this group is due to the A-PRF osteoinductive properties. This means that MG-63 cells, under different concentrations of A-PRF, exit the proliferation phase for a short period of 72 hours and enter the differentiation phase; however, in the L-PRF group, their entry into the differentiation phase is prevented because of increased proliferation. 

According to the results of previous studies [ 1 , 23 , 29 ], the results expected from Alizarin Red staining were the observation of calcium nodules in the two main experimental groups (L-PRF and A-PRF). However, Fujioka *et al*.’s study [ 14 ] by proving the rich content of A-PRF growth factors compared to other fibrin products, and Kobayashi *et al*.'s [ 16 ] study by showing the higher levels of type I collagen mRNA in A-PRF than L-PRF, justify A-PRF’s early effects on cell differentiation.

The cause of mineralization stimulation of A-PRF at all concentrations might be traced in a three-dimensional structure, the process of fibrin network formation, and the role of leukocytes. The decrease in centrifugation speed and force in A-PRF preparation, results in higher density of blood cells, such as leukocytes, throughout the fibrin clot, whereas in L-PRF these cells are rapidly driven to the lower areas (buffy coat) and are less involved in the structure fibrin clot due to their heavy weight and higher centrifugal force.

Leukocytes are a very rich source of cytokines and growth factors such as bone morphogenetic proteins (BMP), PDGF, and IGF that influence osteoblast differentiation [ 30 ]. Unlike platelets, the release pattern of growth factors by leukocytes is not rapid degranulation; it is initiated by inflammatory and pro-inflammatory cytokines interaction with their specific receptors that occurs over time. On the other hand, delayed alkaline phosphatase activity of leukocytes could also help facilitate the mineralization process of MG-63 cells in 72 hours of exposure to A-PRF extract.

Platelets that are more abundant in A-PRF than L-PRF [ 14 ] are the primary source of three growth factors including IGF (capable of stimulating cell differentiation), VEGF (bone growth enhancer), and TGFβ1 (a mineralization and extracellular matrices synthesizer) [ 31 ]. This might be another reason for the faster occurrence of mineralization in A-PRF and the lack of observation within a week in L-PRF.

Moreover, by allowing the longer time required for mineralization evaluation, the results might be slightly different because the MG-63 cell line does not generally exhibit rapid mineralization [ 32 ]. The characteristics of the presence of both mature and immature osteoblast cell types in MG-63 cells and its heterogeneity with other osteosarcoma cell lines [ 33 ] make it difficult to judge osteoblastic differentiation in these cells. However, the proven effect of PRFs (in general) on cell differentiation cannot be ignored [ 1 , 10 , 23 - 24 , 34 ]. The effect of L-PRF on osteoblast cell differentiation might be delayed, and in a longer period, signs of differentiation in the environment might be apparent. In this group, cells might begin to differentiate after reaching maximum confluency [ 35 ]. 

Osteoblasts require at least 14 days to mineralize, and the use of other cell differentiation assays, such as the evaluation of alkaline phosphatase activity and the presence of type I collagen [ 24 ], will be helpful in a shorter time. In addition, despite the lower expression of bone sialoprotein and osteocalcin in MG-63 cells [ 33 ], it might be possible to resolve this problem by examining these osteogenic markers as well as osteopontin, which is expressed at a higher rate in the late stages of osteoblast differentiation [ 28 ].

In other studies, evaluation of mineralization in the dental follicle and periodontal ligament cells [ 22 ] and dental pulp stem cells [ 29 ] by Alizarin Red staining showed that mineralized nodules were developed seven days after exposure to PRF. Since differentiation is highly dependent on the cell type, it is better to compare the present study with those of MG-63 cell line studies. However, no study has been performed to investigate PRF’s effect on the differentiation of MG-63 cells. Since almost all of these studies reported an increase in differentiation tests over time, further studies are advisable to consider mineralization of MG-63 cells at longer times (at least 14 days).

A-PRF and L-PRF might be used for bone remodeling. However, there is still controversy over the superiority of A-PRF or L-PRF in the conflicting results of studies [ 14 - 15 , 19 ].

Two processes including angiogenesis and collagen synthesis control the wound healing. Platelets are involved in increasing collagen synthesis by cells, bone remodeling, homeostasis, facilitation of fibrin clot formation, and secretion of factors involved in angiogenesis. Leukocytes also play a role in preventing infection by phagocytosis of debris, germs, and dead tissues [ 36 ]. Macrophages are also a source of chemotactic agents essential for the stimulation of angiogenesis [ 31 ] and have a special role in the secretion of growth factors such as TGFβ, PDGF, and VEGF [ 36 ]. These growth factors and hormones regulate bone regeneration by affecting cell differentiation and cell growth regulation [ 28 ]. Moreover, Chen *et al*. [ 37 ], in a systematic review of randomized controlled trials suggests that platelet-rich products enhance wound healing.

Bone formation is strongly controlled by bone and endothelial cell interactions in the healing region. In addition, biological factors like TGFβ, BMPs, FGF, PDGF, and IGF can potentially be used in bone regeneration [ 28 ]. The synergistic effect of BMP-2 and VEGF by stimulating osteogenesis and angiogenesis [ 38 ] and the importance of BMP-2 in osteogenesis are well known. The positive effects of BMP-2, BMP-7, IGF-1, and PDGF on the regeneration of periodontal lesions have been reported, and RUNX2 and BMP have been identified as the most potent regulators of osteoblastic differentiation in mesenchymal stem cells [ 28 ]. 

The role of PRF in the overexpression of bone sialoprotein, osteocalcin, alkaline phosphatase, and RUNX2 genes has been demonstrated [ 39 ]. One of the most crucial growth factors of PRFs is TGFβ, which has been claimed to stimulate BMP activity in the early stages of bone healing. The rich content of PDGF in PRF also stimulates the synthesis and secretion of TGFβ from macrophages and activates this cycle. In fact, PRF indirectly affects bone growth and maturation through cytokines and growth factors [ 30 ].

Currently, no system can mimic all the biological functions of autologous bone grafts. An ideal bone graft is a scaffold with structure and properties similar to the bone extracellular matrix [ 40 ]. Given the positive effect of L-PRF on the proliferation and stimulation of mineralization by A-PRF in pre-osteoblastic cells observed in this study, and the ability of PRFs to maintain bioactive materials and play a role as a scaffold, the concomitant use of both A-PRF and L-PRF membranes might be efficient in areas requiring bone grafting.

Ghanaati *et al*. [ 13 ] in a comparison of these two platelet products has shown A-PRF to have a looser structure with more spaces between the collagen fibers and more cells in its fibrin clot than L-PRF. Besides, the existing cells are more evenly distributed throughout the fibrin clot. However, L-PRF cells are more concentrated in the area near the red blood cells. A-PRF releases higher growth factors than L-PRF, which is attributed to its higher leukocyte content. Dohan *et al*.’s research results [ 19 ] contradict this outcome. In their report, the slow release of PDGF-AB, TGFβ1, and VEGF from the L-PRF membrane at all time intervals was significantly (more than twice) higher than that of A-PRF. BMP-2 was not detected in the A-PRF membrane and was slowly released from the L-PRF for seven days. As observed in the present study, the L-PRF membrane and clot in all the samples were significantly larger and stronger than the A-PRF samples. 

Despite these discrepancies, Clark *et al*. [ 18 ] introduced the A-PRF as a suitable biomaterial for ridge reconstruction because using A-PRF significantly stimulated bone formation compared to freezedried bone allograft. Based on the results, it is likely that these platelet-rich products can be used to regenerate bone defects. Due to the novelty of the A-PRF product, there are limited studies to compare these two products, necessitating further investigations. Further studies on higher concentrations of A-PRF and L-PRF platelet extracts with different and longer times are recommended. 

## Conclusion

Based on the results of the current study, concentrations of 10 and 20% of L-PRF extract after 72 hours had a significant effect on cell proliferation. Moreover, only A-PRF extract in all concentrations was able to stimulate the mineralization of MG-63 cells. Generally, the present study’s results might be explained by the presence of more proliferation and differentiation factors in L-PRF and A-PRF, respectively. 

## Conflict of Interest

Authors declare that there are no conflicts of interest in this study.
